# Bronchial obstruction by tumor embolus of contralateral lung during pneumonectomy: report of a case

**DOI:** 10.1186/1749-8090-8-26

**Published:** 2013-02-19

**Authors:** Dong Kyu Lee, Heezoo Kim, Sang Ho Lim, Young Ho Choi, Hyun Koo Kim

**Affiliations:** 1 Department of Anesthesiology and Pain medicine, Korea University Guro Hospital, Korea University College of Medicine, Seoul, South Korea; 2 Department of Thoracic and Cardiovascular Surgery, Korea University Guro Hospital, Korea University College of Medicine, 97 Guro-donggil, Guro-gu, Seoul, 152-703, South Korea

**Keywords:** Airway obstruction, Bronchial neoplasm, Pneumonectomy, Double-lumen endotracheal tube

## Abstract

Bronchial obstruction due to a tumor embolus of the contralateral lung during pneumonectomy is an uncommon and fatal complication. According to previous cases, a bronchial balloon of double-lumen endotracheal tube (DLT) could prevent a dislodged tumor from traveling to the contralateral lung. We experienced a tumor embolism from the bronchus with cancer to the other bronchus despite applying DLT. A 59-year-old male with endobronchial lung cancer underwent a left pneumonectomy. One-lung ventilation was established by the right-sided DLT. After a left bronchial division, a sudden increase of peak airway pressure and reduction of the expired tidal volume to 50 ml was observed. Intraoperative fiberoptic bronchoscopy showed a near total obstruction of the right main bronchus due to tumor emboli. It was not possible to remove the tumor embolus through bronchoscopic suction and forceps. Therefore, we reopened the left bronchial stump and successfully extracted tumor embolus under bronchoscopic guidance.

## Background

Surgery is one of the curative treatments for patient with primary lung cancer, but pneumonectomy is regarded as a high-risk procedure owing to various complications during peri- and postoperative periods. Because pneumonectomy is a high-priority indication of one-lung ventilation, various methods of lung separation could be applied. However, in the case of pneumonectomy due to endobronchial tumor, lung separation methods using single-lumen tube or bronchial blockers failed to prevent bronchial obstruction of the ventilated lung due to an embolic tumor during operation and this event caused a life-threateneing complication [1-4]. It is believed that an inflated balloon of DLT could protect embolic tumor dislocation into the ventilated lung. We reported a case with tumor emboli dislocation during pneumonectomy even under complete separation between both bronchi by DLT. We regarded that a tumor dislocation to a ventilated lung can occur due to an aggressive manipulation of the contralateral lung during the operation despite the use of DLT.

## Case presentation

**Figure 1:**
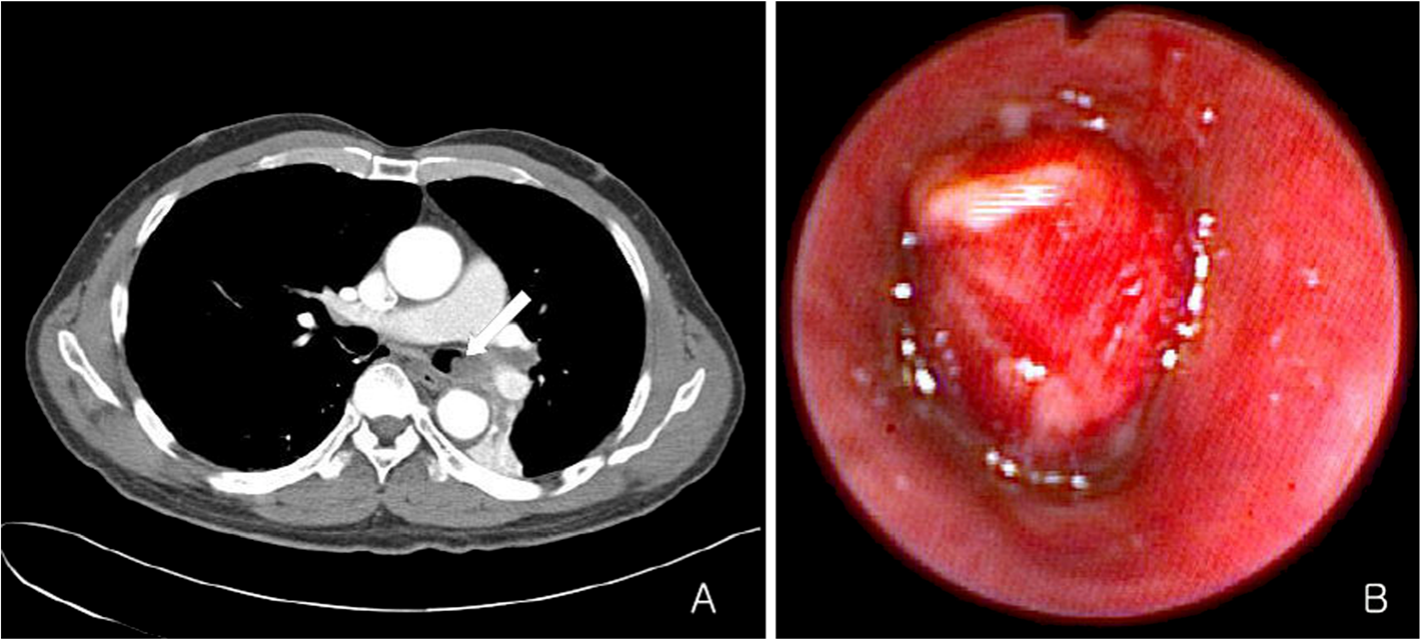
An endobronchial tumor (arrow) in the distal left main bronchus was shown in the chest computed tomography (A) and bronchoscopy (B).

A 59-year-old male patient with a non-small cell lung cancer was scheduled for a left pneumonectomy. Chest computed tomography (CT) revealed that an endobronchial mass was located in the distal left main bronchus to the proximal left lower lobe with complete obstruction, which was consistent with a bronchoscopic examination (Figure 1). Forced expiratory volume in 1 second was 1.83 L in the pulmonary function test and arterial blood gas analyses were pH 7.43, pCO_2_ 49 mmHg, and pO_2_ 76 mmHg at room air.

**Figure 2:**
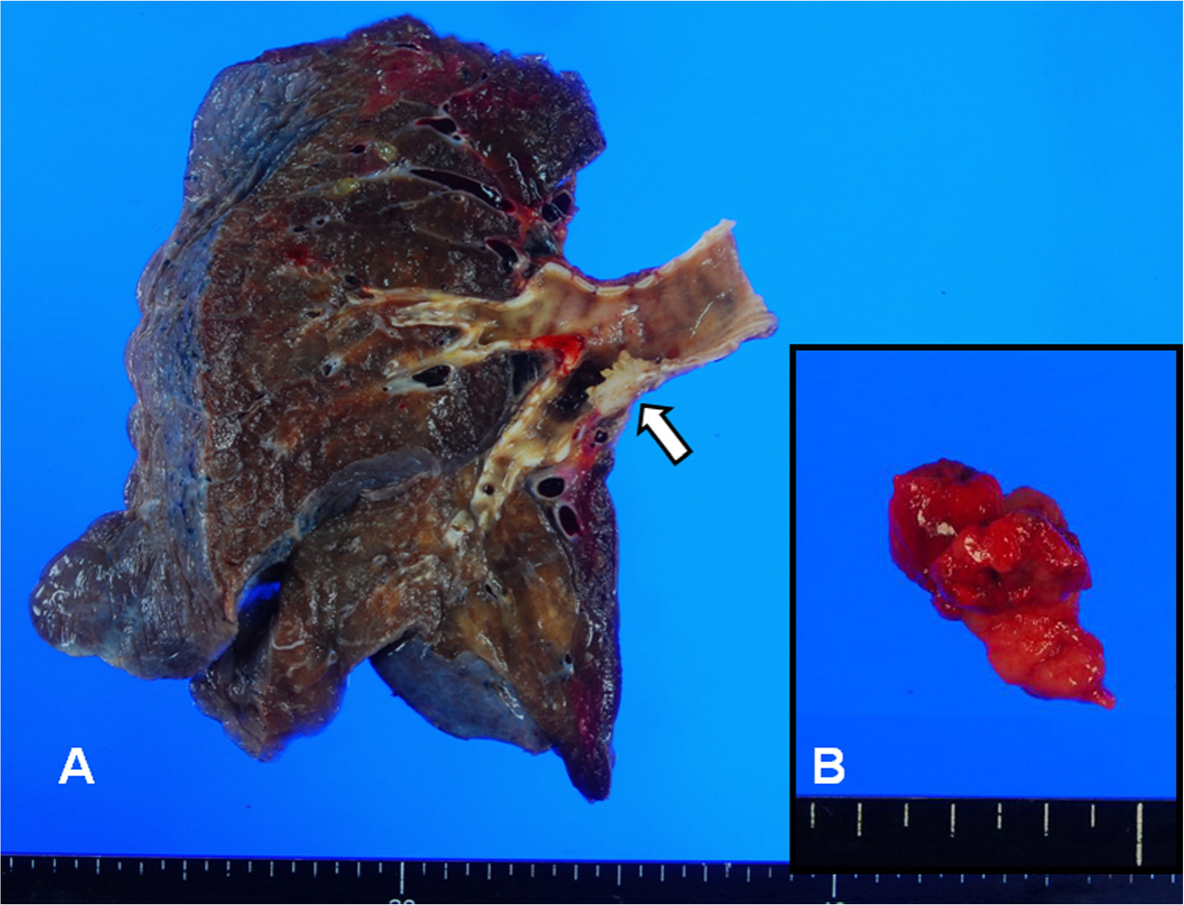
**(A) The gross specimen showed that the tumor was located in the distal left main bronchus.** (**B**) The tumor emboli (size: 1.7 × 1.6 × 0.5 cm) was removed from the right main bronchus.

When he arrived at the operation room, oxygen saturation was maintained at 96% at room air and was increased to 100% immediately after preoxygenation maneuver. A 37-Fr right-sided DLT (BronchoCathTM, Mallinckrodt Medical Inc., Athlone Co., Westmeath, Ireland) was placed for lung separation under fiberoptic bronchoscopy (FOB, LF-2, flexible fiberscope, Olympus, Tokyo, Japan). During two lung ventilations with a tidal volume (TV) of 500 ml, the peak airway pressure (PAP) was 26 cmH_2_O. We performed FOB and confirmed that the bronchial balloon was in a proper position. Next, right-sided one-lung ventilation was initiated with clamping the tracheal side of the DLT. Ventilation was maintained with FiO_2_ 100%; TV 400 ml; respiration rate 15 per minute and PAP was maintained at approximately 25 cmH_2_O. After 15 minutes, the arterial blood gas analyses were pH 7.54, pCO_2_ 29 mmHg, and pO_2_ 516 mmHg. Approximately 3 hours after the start of the operation, and shortly after the entire left lung was extracted by a division of the left main bronchus, the PAP suddenly increased and reached up to 50 cmH_2_O and the expired TV was below 50 ml. We suspected a cuff malposition, and immediately performed FOB. The bronchial cuff was in an adequate position without cuff herniation, and the cuff volume was enough to seal the right main bronchus, but we found a tumor embolus at the end of the bronchial side tube, which produced a near total obstruction of the right main bronchus. We promptly tried to extract the mass by performing suctioning; it was useless. In order to gain time for the next procedure and to prevent hypoxemia, we introduced bronchoscope, which have a suctioning channel through the left-sided lumen of DLT, passed over the embolic tumor mass to place the tip of bronchoscope on distal part of the right main bronchus and supplied oxygen through suctioning channel with high flow (10 ml/min). At this time, manual bagging with anesthesia bag produced various expired TV from 30 to 300 ml. Another bronchoscope (LF-DP, flexible fiberscope, Olympus, Tokyo, Japan) and a basket (FG-51D, disposable grasping forceps, Olympus, Tokyo, Japan) were applied to extract the embolic tumor mass through the right-sided lumen of DLT. However, it was not easy to retrieve the mass using a basket or suctioning, because the nature of the embolic mass was too soft. During these attempts, vital sign was remained blood pressure 100/60 mmHg, heart rate 90/min, and SaO_2_ 92-95%. About 20 minutes later, SaO_2_ gradually dropped toward 85%, and eventually, blood pressure decreased 75/50 mmHg. Because bronchoscope itself could be a major obstacle to a hypoxemia-prone patient, we abandoned FOB and removed one bronchoscope, which was introduced later. Vital sign was corrected with small dose inotropics and fluid resuscitation and SaO_2_ recovered around 98% with manual ventilation with anesthesia bag. We decided to extract the embolic mass through the left main bronchial stump under the vision of a flexible fiberoptic bronchoscope. DLT was withdrawn until the tip of the bronchial side tube reached above the carina, and the operator reopened the left main bronchial stump, and removed the embolic tumor mass with curved long forceps (Figure 2). Next, we advanced the DLT with the guide of a bronchoscope towards the right main bronchus, and the one-lung ventilation was restored. Duration of episode was about 30 minutes. At the end of the anesthesia, the patient was extubated safely with adequate spontaneous ventilation.

## Conclusion

The embolic tumor obstruction of the contralateral bronchus during a pneumonectomy is a rare but catastrophic complication. Only a few cases have been reported regarding this complication, and the results varied from complete recovery to death [1-9]. In most reported cases, a single-lumen endotracheal tube was used, but this does not ensure lung separation [1,5,6]. When the bronchial blocker, including the univent tube, was used, the balloon of the blocker should be retracted to facilitate the bronchial suture, which causes this type of accident [2]. Even though a double-lumen tube was applied, an embolic tumor obstruction occurred when the tube exchanged to a single-lumen tube or extubation at the end of anesthesia [3,7]. However, during the operation, most authors believe that an embolic tumor dislocation can be prevented when the balloon was located in the opposite side of the main bronchus using a DLT [1-4]. At the same time, we experienced an embolic tumor obstruction of the opposite main bronchus during the operation without any manipulation of the bronchial balloon.

**Figure 3:**
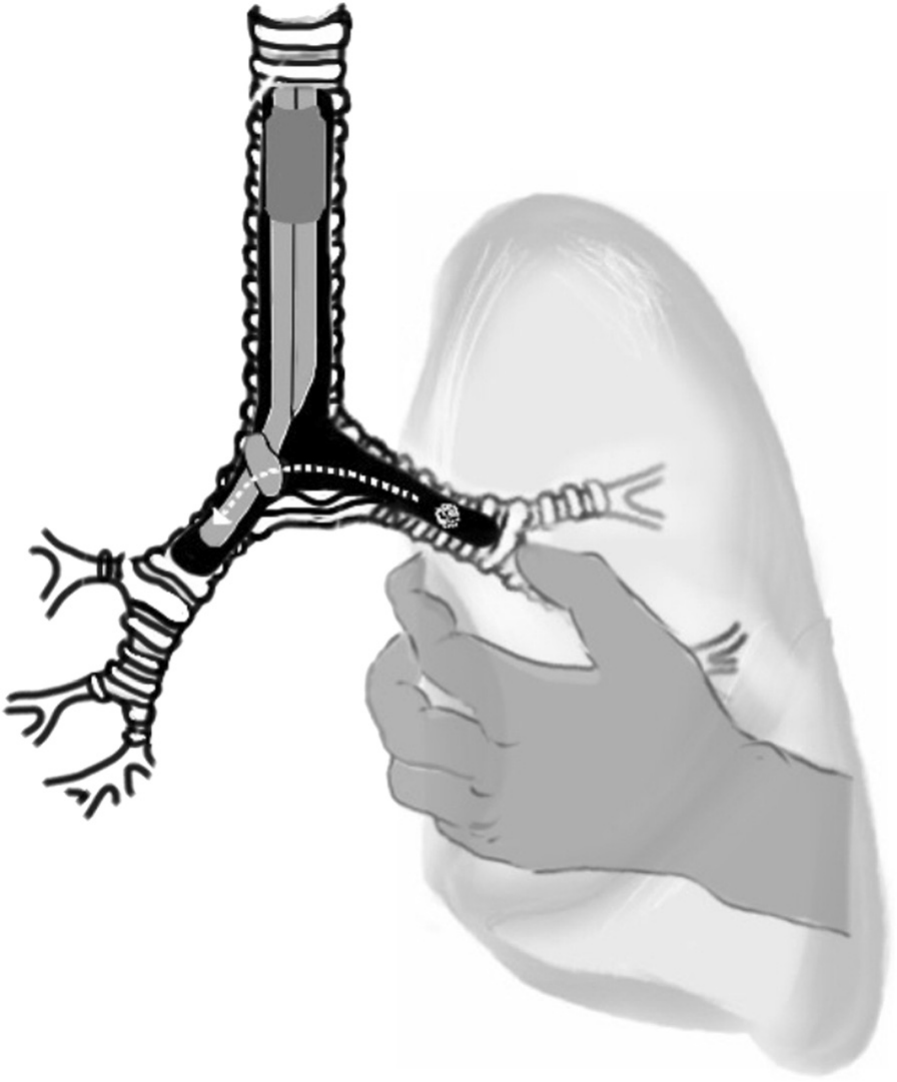
Schematic figure of the left endobronchial mass moving across the bronchial balloon to the right main bronchus during a surgical manipulation around the hilar area.

Most reported cases of embolic tumor obstruction were squamous cell carcinoma or carcinosarcomas, which are known to be friable tumors [6,10]. In this case, the pathologic was pleomorphic carcinoma, which was soft, sticky, and easily compressible. In addition, repetitive partial dislocation of the bronchial balloon by surgical manipulation around the hila area could make the tumor mass to cross over to the bronchial balloon (Figure 3). Therefore, the embolic tumor obstruction can happen at any time during pneumonectomy even if we use DLT.

When bronchial obstruction by embolic tumor mass occurred, the best choice of treatment is depend on the process of surgery, patient position, size and number of embolic mass, site of obstruction, method of maintaining one-lung ventilation, and so on. Extraction of embolic mass with FOB requires experienced person and adequate equipment. Because of the limitation in the instruments with FOB, rigid bronchoscope could be a better choice of treatment. However, in this case, because the patient was in the right-decubitus position and left chest wall remained opened, rigid bronchoscopy was not easy to perform. Therefore, surgeon reopened the left main bronchial stump, and removed the embolic tumor mass with curved long forceps under the vision of a flexible fiberoptic bronchoscope.

It is important to recognize bronchial obstruction as early as possible with continuous monitoring. Any changes regarding the PAP, SaO_2_, TV, end-tidal CO_2_, and other parameters for ventilation monitoring should be continuously checked and evaluated. Frequent FOB is also highly recommended when bronchial obstruction is suspected, especially before and after a bronchial resection.

## Consent

The written informed consent was obtained from the patient for publication of this case report and all accompanying images including CT, X-ray and bronchoscopic findings. A copy of the written consent is available for review by the Editor-in-chief of this journal.

## Abbreviations

DLT: Double-lumen tracheal tube; CT: Computed tomography; FOB: Fiberoptic bronchoscopy; TV: Tidal volume; PAP: Peak airway pressure.

## Competing interests

The authors declare that they have no competing interests.

## Authors’ contributions

HK Kim and YH Choi performed surgery and DK Lee performed general anesthesia and provided one-lung ventilation during operation. HZ Kim and SH Lim participated in bronchoscopic mass extraction and maintaining ventilation during procedure. HK Kim and DK Lee were major contributors in writing the manuscript. All authors read and approved the final manuscript.
